# Managing Knowledge Integration in a National Health-Care Crisis: Lessons Learned From Combating Sars in Singapore

**DOI:** 10.1109/TITB.2005.847160

**Published:** 2005-06-10

**Authors:** Paul Raj Devadoss, Shan Ling Pan, Shreyan Singh

**Affiliations:** Department of Information SystemsSchool of ComputingNational University of Singapore Singapore 17543 Singapore

**Keywords:** Health-care information systems (HCIS), knowledge, knowledge integration

## Abstract

The outbreak of Severe Acute Respiratory Syndrome is the first severe and readily transmissible disease to emerge in the 21st century. Often one new infection meant tracing of several people to monitor their health conditions as well. In Singapore, several agencies coordinated their efforts to quickly bring the outbreak under control. The current breed of health-care information systems (HCIS) was not sufficient to handle new information-sharing needs during the crisis. In this paper, we take a look at the measures taken during the crisis in Singapore through a knowledge integration perspective. This perspective reveals interesting implications for HCIS.

## I. Introduction

The USE of information technologies (IT) in the health-care industry is often restricted to reducing costs and increasing quality of health care [1]. IT innovations increasingly facilitate better knowledge management processes for better health care [2], which can be achieved through better task focus to knowledge-sharing activities [3]. While cost reduction and quality of health care rightly share a majority of the focus, changes in the world are rapidly exposing us to new potential dangers such as the virus strain that caused the outbreak of Severe Acute Respiratory Syndrome (SARS) in many regions in Asia and Canada, which is potentially only the first such danger in this century [4].

Health-care services are generally collaborative work involving many stakeholders [5], [6]. The management of health-care emergencies is more so, and is a complex process which has to be tackled on various fronts [7], [8]. To compound the process, the strain was a new virus, leaving people in the dark as to how the virus spreads and what measures may or may not be effective. In Singapore, early measures put in place effective mechanisms to detect suspected cases of SARS, isolate, and treat them to prevent escalation of the crisis. Immediately upon realizing close contact as a cause for transmission of the disease, many initiatives were taken to manage the crisis. Contact-tracing of confirmed SARS cases was carried out, suspected cases were quarantined, and social support was provided to those affected, through various grassroots agencies. Various agencies from the Ministry of Health (MOH) to the Immigration and Checkpoint Authority (ICA) were involved in attempting to contain the spread of the infection. This process meant effective coordination and dissemination of current and useful information to various agencies. Information flow, key to effective management of a crisis [7], was also a key issue in the SARS outbreak.

Hospitals and various health-care institutions were the battlegrounds where management of this crisis had to be effected. Health-care information systems (HCIS) had to share information effectively to help manage this battleground. Though Singapore already utilizes several strategic applications of IT among health-care institutions [9], the SARS outbreak posed new challenges which needed to be addressed. Since existing HCIS are generally not designed to cater to new needs [1], when the crisis escalated, Singapore developed and deployed an information system in two weeks to help manage data and various related operations during the crisis. In Toronto, where information flow was also recognized as necessary to manage the SARS outbreak, such rapid development and deployment of information systems could only be hoped for and post-its were used to manage contact-tracing operations [10].

In this paper, we narrate the crucial role of IT in coordinating information flow in managing the crisis in Singapore involving several agencies, to draw useful lessons for HCIS through a knowledge integration perspective. Knowledge integration is the ability of an organization to integrate specialist knowledge. Grant discusses efficiency, scope, and flexibility as three characteristics of knowledge integration [11]. These three characteristics, in the context of health-care crisis management, reveal the need for HCIS to be able to share information efficiently across several agencies, and integrate at least basic data to enable smooth information flow.

In Section II, we discuss some literature on IT in health care to illustrate the primary focus of current breed of information systems and their usage. Section III describes research methodology and Section IV narrates the case. In Section V, we illustrate some useful lessons form the analysis of the case, followed by Section VI, where we discuss some implications for HCIS and future research.

## II. Literature Review

### A. IT in Health Care

Diverse IT applications in health care are collectively referred to as HCIS, including applications from diverse fields such as medicine, computer science, management science, statistics, biomedical engineering, among others [12]. Even though the health-care industry lags behind lead adopters of IT such as the financial and airline sectors, research findings illustrate that resources committed to IT by health-care institutions have increased from approximately 2% of revenues to about 5%–7% [13]. Developments in the Internet, enterprise-grade decision support, and mobile technologies underpin many of the advancements in health-care IT [14]. However, the implementation of disease-related groups and disease-management programs to manage health-care costs and medical insurance is leading to greater need for advanced IT in health care. At the broadest level, health-care institutions derive two types of benefits from the use of IT, namely, *reduced costs* and *increased quality* of service delivery [1].

Traditional health-care information systems focus on automation and integration of administrative tasks such as billing and payments collection [1]. With the advent of enterprise resource planning (ERP) systems and complex supply chain management solutions, these administrative IS are increasingly perceived as means to reduce costs and increase efficiency by integrating cross-functional data and streamlining procurement tasks. The health-care industry has shifted from using proprietary and expensive electronic data interchange solutions to embrace e-marketplaces for procurement of supplies. E-marketplaces provide a number of services cost-effectively, including online catalogues, ordering, auctions, and inventory management [15]. Prominent e-marketplaces include the Global Healthcare Exchange and Neoforma.

Clinical information systems (CIS) represent another category of HCIS that is gaining widespread acceptance in the health-care industry [1]. CIS are aimed at improving quality and management of health care, through a variety of technology applications ranging from decision support systems (DSS) to patient relationship management (PRM) solutions [14]. Electronic health records (EHR) contain all clinical and administrative information related to a patient as a basis for all health information systems. DSS support clinical decision making by providing information about diagnostic procedures to physicians and alerts to telemedicine patients about preventive care [1], [16]. Artificial intelligence, neural networks, and fuzzy logic techniques are used to develop such systems [12]. Data mining and PRM solutions leverage data in an institution's ERP system or in external data sources such as MEDLINE and CliniWeb to provide better solutions to health-care problems as well as to forecast needs and tailor treatment to individual patients based on previously stored information. Such personalization solutions necessitate the creation of computer-based patient records containing relevant clinical, administrative, and biographical data of a patient [12], [14]. The trend toward development of universal electronic patient records, in turn, demands the integration of these two categories of information systems.

### B. Knowledge Integration

For the purposes of this paper, we adopt Nonaka and Takeuchi's definition of knowledge as “justified true belief” [17]. Knowledge exists both in the individual and the collective [18]. Organizational knowledge is embedded in its employees, systems, culture, routines, policies, and practices [19], [11], [20]. Knowledge management is the process of identifying and leveraging collective knowledge in an organization to enhance its ability to compete [21]. The importance of organizational knowledge management is underscored by the increasing attention that the subject has received from both researchers and practitioners [22].

Drawing on the sociology of knowledge, Alavi and Leidner view organizations as social collectives and knowledge systems and present a framework of knowledge management that comprises four sets of socially enacted processes [19]. These processes are 1) knowledge creation, which involves development of new content or replacement of existing content [23], 2) knowledge storage/retrieval, also referred to as organizational memory residing in expert systems, databases, and documented procedures, 3) knowledge transfer, which involves communication processes and information flows to move knowledge to locations where it is needed, and 4) knowledge application, which involves the creation of new knowledge through integration and application of existing knowledge. Knowledge integration is a key aspect of the knowledge application process [24], and is perceived as a source of organizational competitive advantage [11]. Effective knowledge application premeditates an organization's ability to sense, interpret, and respond to new environmental opportunities and threats [24].

Knowledge integration is defined as a continuing collective process of creating, expressing, and redefining shared beliefs through social interaction of organizational members [22], [25]. In addition to enhancing an organization's competitiveness, effective knowledge integration enables coordination and synthesis of cross-functional expertise and activities, which, in turn, serves as a mechanism to refine and create knowledge [25].

Grant identified three characteristics of knowledge integration, namely, efficiency, scope, and flexibility of integration within an organizational setting [11]. Efficiency is the ability to communicate and use knowledge from different pools of expertise [11]. Scope is the breadth of specialized knowledge an organization can access [11]. Flexibility is the ability to access new knowledge and innovate in an organization [11]. Though Grant's discussion on knowledge integration is based on organizational capability development [11], we suggest that these basic characteristics of knowledge integration are relevant in all contexts of knowledge application.

In this paper, we study these three characteristics of knowledge integration in the context of managing a health-care crisis. Efficient flow of information is necessary in managing an outbreak [7]. Also, the ability to integrate knowledge from various resources, and deliver innovative solutions that meet unique requirements is an important part of managing a health-care crisis. In the rest of this paper, we narrate the management of a health-care crisis to demonstrate important lessons in the use of IT in health-care management.

## III. Research Methodology, Analysis, and Data Collection

Qualitative research emphasizes processes and meanings that are not easily quantified. It stresses how social experiences are created and given meaning [26]. According to Yin, case study research is most appropriate in scenarios where the research question is exploratory in nature and focuses on examination of contemporary events that occur beyond control of the investigator [27]. Interpretive methods of research are “aimed at producing an understanding of the context of the information and the process whereby the information system influences and is influenced by the context” [28]. Hence, an interpretive case study methodology has been adopted for this study. Further, Klein and Myers discuss principles of interpretive research, providing useful guidance to the conduct of this study [29].

Data for this study was collected from multiple sources for triangulation [30], and is part of a study on the management of the crisis and the role of IT. Eighteen personal interviews were conducted to obtain a breadth of information, opinion, and experience [31]. All interviews were transcribed and analyzed, and each interview lasted an hour and a half on average. The interviewees were from the Defence Science and Technology Agency (DSTA) who provided all technological support during the crisis, and coordinated extensively with all agencies at all levels. Data collected during personal interviews focused on understanding the participants’ understanding of the crisis, their contribution, experiences, and perceptions. Due to extensive coverage of the outbreak in Singapore, abundant secondary data were also collected.

Data collection and analysis are hard to differentiate in qualitative research [32]. The data is iteratively analyzed with theory [33]. Open coding the data in accordance with grounded theory [34] iteratively with transcribed interview data, secondary data, and observations allowed some themes to emerge from the analysis. This analysis was informed by a review of literature on knowledge management and knowledge integration and IT in health care. Grant's discussion of three characteristics of knowledge integration was used to organize the findings to draw implications to HCIS [11].

DSTA, the lead agency managing the technology support during the crisis, was formed by the Government of Singapore as a statutory board under the Ministry of Defense (MINDEF) in March 2000. DSTA is responsible for implementing defence technology plans, managing defence research and development, acquiring defence material, and developing defence infrastructure for MINDEF. Apart from its defence technology support, DSTA's development work in IT involves various applications that enhance command and control and daily operations of Singapore's Armed Forces. Through its various operations, DSTA has expertise in providing enterprise systems solutions, knowledge engineering, mobility, military command and control, and e-government systems [35].

## IV. SARS Outbreak in Singapore

### A. Origins of the Outbreak

In February 2003, several people were diagnosed with a severe form of pneumonia in the Guangdong province of China. The infection spread to Vietnam, traced to a traveller, returning from China and Hong Kong, during the later part of February. Several members of the hospital staff were infected and became critically ill. On the 11th of March, the Prince of Wales hospital in Hong Kong reported several infections and was monitoring several staff with a breakout of fever and respiratory problems.

On the 12th of March 2003, the World Health Organization (WHO) issued a global alert on the outbreak of a severe form of pneumonia. Following the alert, Singapore's MOH issued a national alert, requesting doctors to be on the lookout for flu-like symptoms and respiratory problems in patients. It also requested travellers from these regions to consult doctors immediately upon developing any flu-like symptoms. MOH was also monitoring the health of three patients who developed these symptoms and had recently returned from Honk Kong. While two were discharged upon recovery, one patient was under observation and recovering. MOH did some contact-tracing and monitored those who had been exposed to the patient.

### B. Spread of the Infection in Singapore

Named SARS by WHO, soon more cases of the disease surfaced, further complicating the spread of the infection. Tracing was also increasingly difficult with over 95 infected patients and 52 discharged. Apart from tracing contacts with suspected carriers, monitoring was established at the airport to help screen passengers as they arrived in Singapore. ICA worked in conjunction with the National Environment Agency (NEA) to help trace passengers who needed to be quarantined such as in the case of the fourth index patient (primary carriers who infect others) who flew into Singapore with symptoms of SARS. The Ministry of Education (MOE) was working with the educational institutions to help trace contacts within institutions and implement screening measures to identify flu-like symptoms early.

**Fig. 1.**
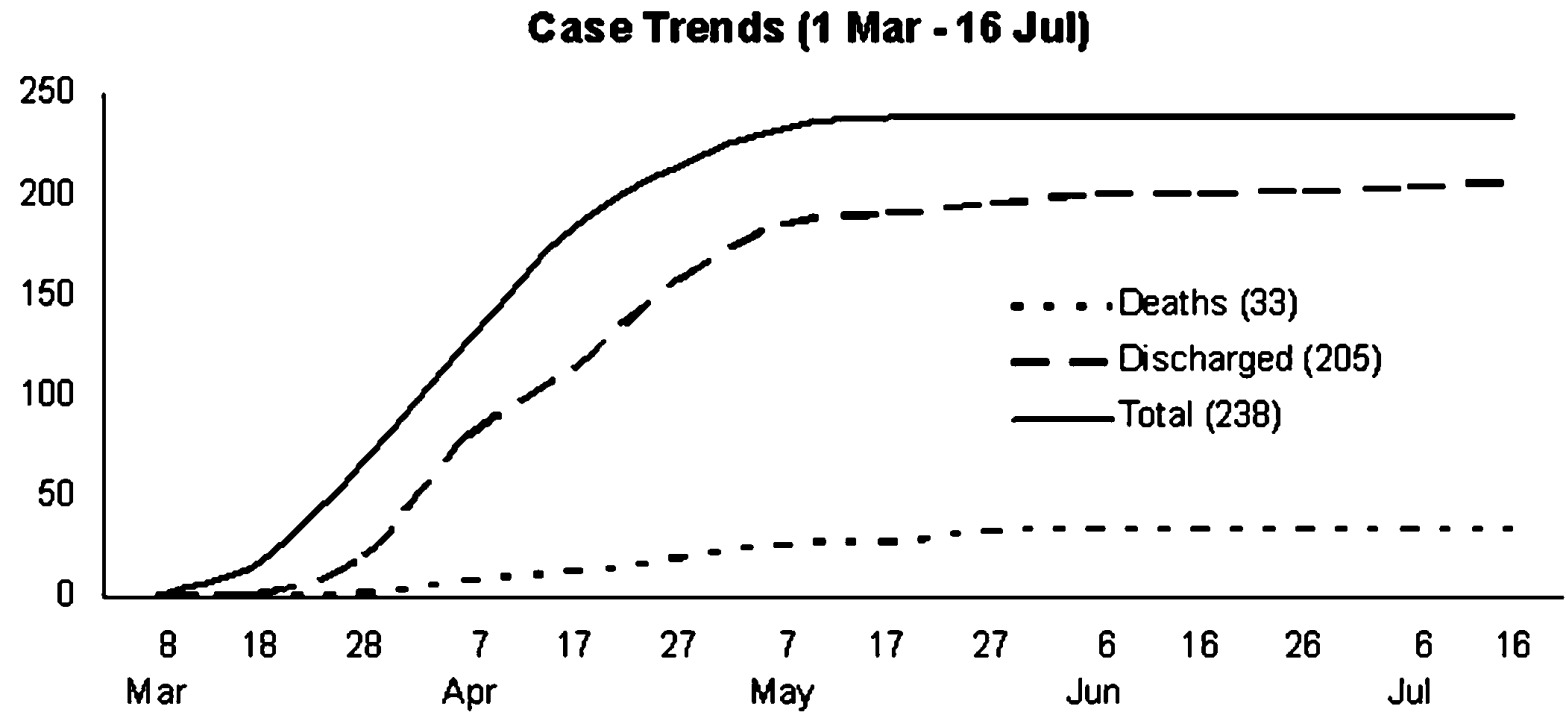
Spread of SARS infection in Singapore (from www.moh.gov.sg/sars).

Increasing index cases and the need to trace and quarantine all contacts of known infected patients led to increased strain on resources. Despite the nation-wide measures taken to help identify early symptoms and isolate patients, the cycle of infection was not broken and every day patients trickled into hospitals with SARS symptoms. On the 19th of April, a new chain of events began, which took the SARS crisis to a new level in Singapore. A cluster of new infections were discovered, and one of them worked in the Pasir Panjang Wholesale Market in Singapore. The new cluster meant the entire wholesale market had to be shutdown and over 700 people contacted to check for symptoms of SARS. Some infected patients had visited several general practitioners and several *sinsehs* (doctors in Chinese medicine) before visiting a hospital. Fig. 1 represents the numbers of infections and fatalities during this outbreak in Singapore.

Contact-tracing had to deal with identifying all visited places, and contact each person to monitor their health. The decision was to play it safe and quarantine even a large number, rather than risk letting potential patients slip through. Close contacts were quarantined and others monitored for appearance of SARS symptoms. All stall holders in the market and their employees were quarantined. Everyone who visited the wholesale market between the 5th and 19th of April were requested to report to the MOH or a hospital to be screened for possible infection.

New cases were trickling into the hospitals now, mostly related to the wholesale market. Contact-tracing was in full swing, but with the escalation of contacts to be traced, the system was under severe pressure. In Section IV-C, we describe the contact-tracing procedures followed by the NEA with the help of MOH in Singapore and the armed forces.

### C. Contact-Tracing

During the early outbreak of SARS, the hospital staff traced contacts. This was soon becoming cumbersome since the medical staff was under pressure catering to medical needs of continuously trickling cases and increased precautions during medical care provision. MOH established an operations center together with the NEA to conduct contact-tracing operations. Officers from the NEA managed data on Microsoft Excel-based spreadsheets to monitor data and obtain contact details. Information gathered from contacts was keyed into those spreadsheets. One of the users commented that the spreadsheet was “powerful and caters to all their needs” because it allowed freeform data entry.

Reports were prepared based on such information to update MOH on the status of contact-tracing and issuance of Home Quarantine Orders (HQO). MOH contracted CISCO, a statutory board specializing in security services in Singapore, to help in issuing and verifying compliance of HQO. Thus, the complete process of identifying a suspect case, the patient's contacts, and issuance of HQO involved four major agencies, namely, MOH, the hospitals, the NEA, and CISCO. Other agencies were also involved in providing social support such as counselling, food delivery, etc.

When a patient was identified as a suspect SARS case, this information was passed on to the contact-tracing operations center. The MOH did its own follow-up and sometimes obtained information from other relevant agencies (e.g., MOE provided some information on a student's school, etc.). This information was consolidated and sent to the operations center, which was in the process of tracing contacts. This information was consolidated as a complete list of people to be issued with HQO and delivered to CISCO by 8 p.m. every day. CISCO's staff then visited the people on their list and issued HQO. This procedure had to be completed by midnight or early morning in order to ensure compliance with the HQO immediately. Nonissuance or noncompliance only meant a potential increase in people to be traced.

### D. Scaling Contact-Tracing Operations

Escalation of the outbreak and the need to contact and trace an entire wholesale market in Singapore prompted MOH to establish contact with the MINDEF to set up a bigger operations room for the growing contact-tracing operations.

On the 24th of April 2003, four days after the Pasir Panjang Wholesale Market incident, DSTA was requested to provide their assistance to establish an operations room for contact-tracing. The initial IT infrastructure centered on setting up 120 stations with e-mail, file, and print services and Internet connectivity to the government's e-mail systems. This was established within 48 hours. This capacity was subsequently increased to 250. Due to the nature of the SARS viral transmission, video conferencing became critical connecting MOH and MINDEF. A support team was established, which operated in shifts to provide technical support to the operations center.

The chief information officer (CIO) of DSTA oversaw the operations and suggested that the infrastructure would still not help MOH scale-up its operations because their business process was not sufficiently streamlined. “Most of the information was in hardcopies or on spreadsheets with unstructured data. It would be hard to do any sort of analysis based on that data,” reported an informant. Hence, DSTA suggested the use of an information system to cater to the needs of information coordination and flow, thus making the process of tracing contacts efficient.

### E. Building Case-Management System (CMS)

DSTA had two weeks to gather information on all processes in tracing contacts, identifying their linkages, and issuing quarantine orders. The system had to be developed to meet the requirements as anticipated by DSTA. To begin this process, DSTA began by assembling a team that was experienced in network technologies, database administration, and systems development. Employees were invited to join the development team, and asked to drop all existing projects to complete the CMS in two weeks.

Most of the invited employees began work immediately, recognizing the critical nature of the assignment. The national crisis motivated most team members to contribute their efforts to the project. Another reason cited by developers was the challenge of having to develop a system in such a short time, and gathering development requirements as the system was being written. “It was like being in some kind of extreme programming competition!” suggested a programmer.

The project team was assembled, and the nearest system to contact-tracing requirements was identified to help jump-start the development process. DSTA had previously developed a casualty management system for the Singapore Armed Forces. This system was identified as the closest to current requirements and the project managers had prior experience in developing that system. However, this system was insufficient to manage tracing operations, which required analysis of linkages among the infected patients and their contacts. To help in this process, another government agency provided the DSTA with software to study cross-relationships among a set of people.

**Fig. 2.**
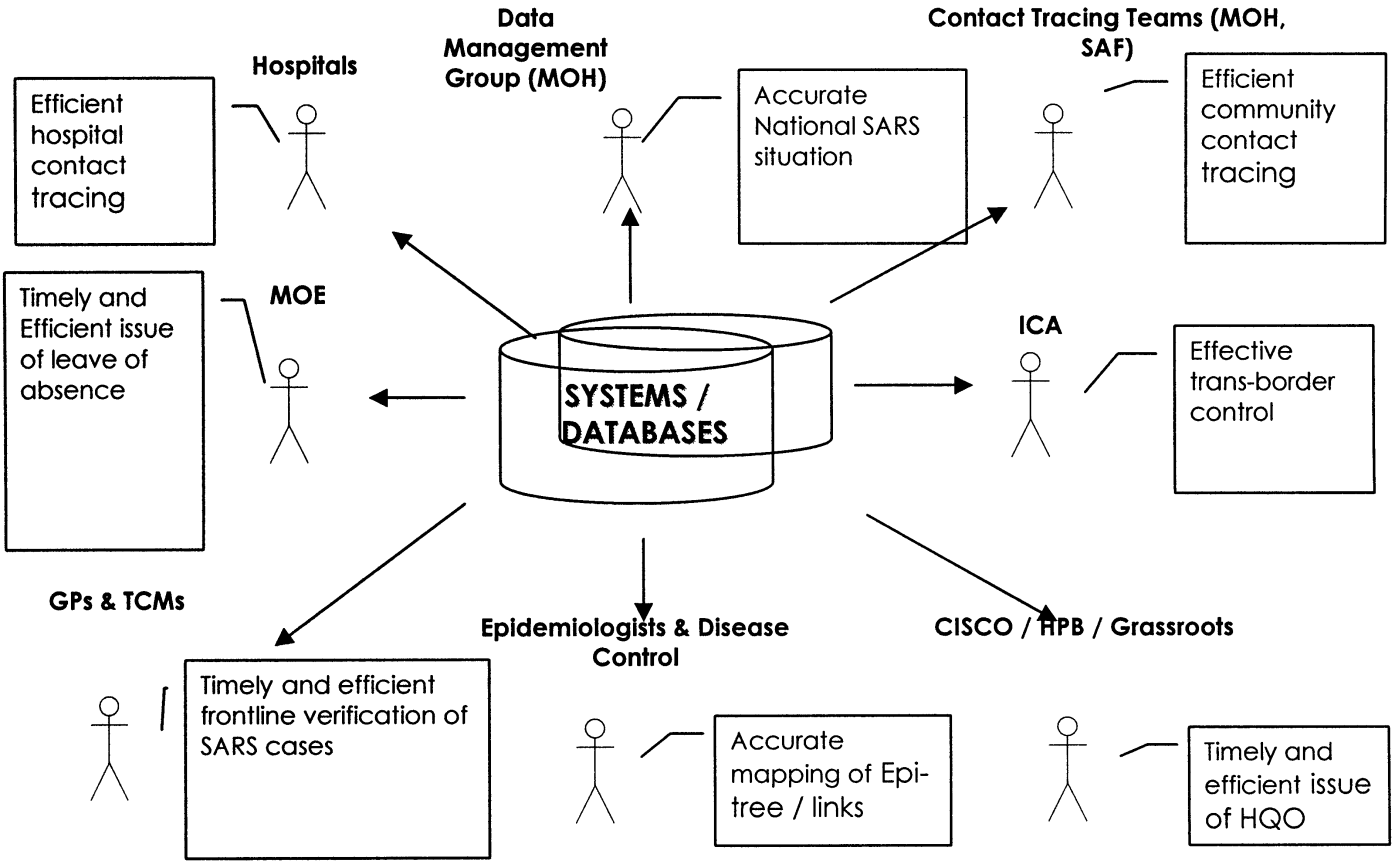
Information management needs that were identified for CMS.

The team quickly went about setting out other requirements for CMS, such as data sources, formats, security, and reports needed from the system. There were no established procedures in the operations room, since data management up to now had been through spreadsheets and individual practices to monitor and manage tracing operations. “People at the operation center had no time to talk to us; they would give us the data and we had to figure out the details. Sometimes they didn't know the complete process!” said a manager. The development team had to identify possible requirements, suggest ways to synchronize their contact-tracing operations, and gather sources of information and user interface layouts. Fig. 2 represents the information sources for contact-tracing operations. Most of these agencies also needed to interact with the system to effectively manage the crisis.

The primary task was to identify sources of data to identify people and their contact information. Sources of information ranged from hospitals, MOE, and MOH, to general medical practitioners and traditional Chinese medicine practitioners. Information from all sources had to be collected into a database, from where the system would perform a case management should someone be identified as suspected or confirmed SARS patient. This database was to be the reference database with as much contact information as could be gathered. In addition, a SARS case-management database connected confirmed cases with suspected and probable cases, to identify potential SARS patients and monitor their health status. This SARS database could also be used to provide exit control with immigration authorities (to prevent infected patients leaving the country, provided by Singapore as a social commitment to the region) or with MOE (to isolate students who inadvertently attend schools when they should be quarantined). The databases together were interfaced through CMS and a link analysis system to help in the entire contact-tracing operations.

The reference database required data to populate the system. It was ineffective with no data to look up contact information. While DSTA worked with other agencies of the government to populate the database with useful information, technical issues centered on inconsistent data formats, incomplete or outdated information. Inconsistencies had to be resolved and data loaded into the reference database. However, that was a small concern compared to obtaining the data itself. “When one agency offered their contact data for the reference database, they cautioned me that the data was at least three months old. But I was ecstatic because back then I had no data and any data was better than that!” said the CIO.

### F. Contact Data From Hospitals

Hospitals were an important zone in the battle against SARS. Patients there had to be isolated to prevent further infection, and at the same time, effective medical care provided to those with SARS as well as others in need of other medical services. Every time a patient was identified as a SARS patient, contact-tracing had to be carried out within the hospital to trace the movement of the patient and monitor the health of relevant staff and other inmates. This was a time-consuming process, but it was critical to the efforts in containing the spread of the virus. Most infections arose from index cases who returned to Singapore from travel to the SARS-infected regions in South East Asia.

The data gathered at such contact-tracing operations were often ineffective due to lack of knowledge on such procedures. Also, there was immense strain on the medical staff at hospitals due to procedures put in place to deal with the crisis. In some of the data gathered there, fields were incomplete, or lacked basic information to conduct meaningful contact-tracing. However, the hospital staff, with their limited technical expertise innovated through the use of Microsoft Visio to plot the linkages to other contacts. Technology savvy doctors helped establish initial contact-tracing data systems with spreadsheets to help trace patients and their contacts.

When DSTA was invited to help MOH with the technical infrastructure, CIOs of health sector agencies had agreed to try a system to tag patients at accident and emergency (A&E) departments of the hospitals since most patients reported to A&E departments when symptoms appeared. One hospital, which was not affected by SARS, ran a trial system with radio-frequency identification (RFID) tags to help trace movements of the patients. DSTA coordinated the trial with a private sector vendor specializing in RFID technologies. Even prior to the outbreak, some hospitals were in the process of trying out RFID systems to help monitor patient movements within hospitals.

### G. Implementation

Development and implementation of CMS was completed in two weeks. However, implementation of CMS and conversion of data from spreadsheet-based systems to the new database system highlighted some interesting experiences. The operations center staffed by NEA officers had developed multiple data formats on the spreadsheets. This meant that the new system had virtually 200 different formats to resolve, making data conversion a difficult process. This was because users were using spreadsheets forwarded by MOH or hospitals to trace contacts. This led to varying formats, and clubbing of several fields in a single cell making it difficult to split the data. Populating the SARS case-management database was, thus, a challenge. The database team set about resolving these issues and populated the database with the case data.

The implementation team worked on parallel implementation to ease the system to the working environment with minimal disruption to tracing operations. Parallel implementation helped implementers convert data into the new system, as well as train users on system usage without disrupting on-going tracing operations. Intensive system testing was also carried out due to the importance of data accuracy. After running the system parallel to the spreadsheet-based data entry, the operations center finally switched over to CMS.

After implementation, DSTA tested and supported the system for three weeks, and was satisfied with its stability. MOH then contracted another agency to continue maintenance and development.

### H. Reappearance of SARS

Relentless efforts were on in Singapore to manage the crisis, and provide a safe and healthy atmosphere in Singapore. The SARS virus had an incubation period of ten days. Singapore was working to break the cycle of infection in order to clear two incubation cycles without new infections and, thus, effectively be rid of the virus. On the 30th of May 2003, Singapore was declared clear of the SARS virus by WHO.

On the 9th of September 2003, a new probable SARS case was reported in Singapore. A postdoctoral student was infected while handling virus samples in a laboratory. His fever started on the 26th of August, but was diagnosed as a common viral fever. He had visited doctors several times with persistent fever. Later, within 8 hours of having identified the SARS patient and activating the tracing operations, about 60 people were traced and contacted and some were issued HQOs.

## V. Analysis and Findings: Contextualizing Knowledge Integration in Health-Care Crisis

Organizations value knowledge as a resource, which will give them a competitive advantage by reducing costs, and improving productivity and innovation [11]. In the context of health-care crisis, the objective is to contain and eradicate the crisis. The primary motivation is providing health care to the citizens. However, such a crisis can also affect the economy of the country, and hence, governments have to demonstrate their ability to quickly and efficiently manage the crisis. This is particularly true in countries which rely heavily on international trade and investments, such as Singapore.

Health-care activities are knowledge intensive, with an explosion of health-care knowledge, and increased patient awareness on health care [36]. However, recent events have demonstrated the need for health-care knowledge management to transcend patient care management and include managing health-care epidemics. The SARS virus is only the first of viral epidemics in this century. Even a recurrence of the virus can cause more damage, and impact occurrence of other diseases [4].

The outbreak of communicable disease such as SARS has, thus, highlighted the need for health-care institutions and governments to be prepared for emergencies. Such a crisis may place a new need for sharing, particularly by health-care institutions. In this section, we highlight some of the lessons learned from Singapore's management of the SARS outbreak, through a knowledge-integration perspective for health-care IT.

### A. Efficiency: Providing Communication and Process Structure

Efficiency of competitive advantage depends on how productive firms are in utilizing knowledge stored within individuals [11]. Efficiently integrating knowledge pools in organizational contexts implies competitive advantage [11]. In the context of managing health-care crisis, efficiency of integration implies better management of crisis. Effective, rapid, and integrated flow of information is important to better manage a crisis [7], with task-based focus to knowledge activities [3].

The ad hoc system that was managed by NEA's staff was inefficient but was sufficient to cover the magnitude of the task in early days of the infection in Singapore. However, when the need to scale the operations arose, better infrastructure was felt necessary. DSTA was roped in, which proposed a system that went beyond merely scaling hardware infrastructure, thus providing an efficient mechanism for communication.

DSTA identified all processes from identifying potential SARS infections to the process of issuing and monitoring HQOs. These processes were built into the system, providing structure to help simplify the process of issuing HQOs. Link analysis of suspected infections helped in understanding the crisis better, even making it easier to compile various reports on the crisis.

All agencies were also linked by e-mail through government e-mail systems. This helped in efficient exchange of information, feedback, and suggestions for further actions. “Every night I would go through the e-mails exchanged by various heads in the government and incorporated their suggestions into the system. It was an extremely useful mechanism,” reported the CIO of DSTA. Summaries of other tasks were exchanged among the agencies to keep each other informed on latest developments.

Hospitals were an important source of data through the entire crisis. They had no means to share information efficiently, and used nonstandard formats in sharing information through spreadsheets. DSTA consulted with the users and suggested standardized formats, thus making it simpler for other users. Tracing operations which relied on this data were further saved the tedious data cleaning process, and focussing on the task of tracing potential infections.

The structuring of processes through DSTA's information system helped the management of the crisis in two ways. It helped coordinate information across several agencies, and it also helped in the accuracy of information dissemination. Coordination among all agencies during the crisis became better because of the information system. The system helped streamline information flow across agencies and the issuing of several HQOs for the same person came under control. This also helped reduce errors in reporting the magnitude of the crisis. This was a key factor since accuracy helped in combating the fear psychosis among the public.

It was also suggested that with a system to monitor the processes, the number of quarantined people may be brought down. This is because it is now possible to take a systematic approach to issuing HQOs. This has potential implications for health-care costs during the crisis through reduced social welfare costs in managing quarantined people.

### B. Scope: Multiagency Process Management

Scope is the variety of knowledge that is integrated [11]. The breadth and depth of knowledge integrated contributes to the complexity of the system. Health-care management in emergencies usually involves several agencies from a variety of domains [7], [8], such as care givers in health-care institutions, managers who help manage the crisis (usually MOH), and other agencies which help monitor or deliver crisis-related activities to help contain the crisis. At each agency, the depth and specialization of knowledge varies.

HCIS focuses primarily on administration and patient data management systems [1], including EHR. The outbreak of the crisis posed new challenges to existing systems. The IT infrastructure was advanced and provided immediate e-mail connectivity to all agencies for information exchange. Hospital staff and health officials used e-mail systems to exchange data, coordinated through NEA's contact-tracing operations. However, escalation of events demonstrated the need for an organized information system. The newly developed databases are now a necessary part of health-care crisis management systems. It could be further developed to integrate key information from general medical practitioners on patient symptoms to collect data on national health-care status, thus integrating all health-care institutions and agencies related to crisis management.

The information from the SARS case database was also mapped through a reporting system to help the administrators of the crisis obtain an accurate picture across the nation. The breadth of agencies involved in the use of CMS is presented in Fig. 2. Data from all relevant agencies were centrally coordinated for information-sharing and coordination. The depth of information, however, was constrained by time limitations on the development of CMS. Two weeks were just sufficient to help integrate all these agencies through various interfaces and share basic information on the SARS outbreak and related cases. This created a mechanism with sufficient efficiency in dealing with the needs of contact-tracing operations. This was also seen as primary data that was necessary to cater to the immediate requirements. Data on quarantine and health were monitored for the potential SARS patients in the system.

### C. Flexibility: Proactive National Health-Care Management

Flexibility of knowledge integration is the ability to continuously reconfigure existing knowledge and seek new knowledge to achieve a competitive advantage [11]. In the context of managing the health-care crisis, the issue of flexibility centers on the ability to innovate to accommodate the needs of crisis management, also known as delivering new capability in Grant's discussion on competitive advantage [11]. Crises, by definition, are rare and unique each time [37], and experience is not a necessary indicator of successful management in future [38]. Hence, it is necessary to be able to manage new sources of explicit and tacit knowledge when necessary.

The outbreak of SARS highlighted a shortcoming in the current breed of information systems which cater to health-care requirements. Systems were not geared to an emergency situation, to connect several agencies to coordinate crisis management activities. With an ever present threat of a recurrence of SARS or other outbreaks [4], it is important to develop health-care systems which can support the information and knowledge needs of the crisis management team. Two key issues become apparent in this situation: HSIC capability and the ability to deploy new capabilities at short notice.

DSTA's development team gathered requirements from ad hoc procedures and built a complete information system and a reference database in 11 days, extending the design of an older system. DSTAs systems knowledge was suitably used to reinvent existing applications and develop systems to meet the needs of the crisis. Now a necessary part of health-care crisis management, the usefulness of the system was demonstrated during the recurrence of SARS in Singapore. Within 8 h, about 60 people were traced and contacted. This demonstrates that effective knowledge application premeditates an organization's ability to sense, interpret, and respond to new environmental opportunities and threats [24].

In Singapore, the contact-tracing operations are designed around interfacing multiple agencies. Though tracing operations are a major aspect of the managing the crisis, the sourcing of data for the system is a significant contributor to the successful use of information systems. The system may eventually be used to continuously monitor appearance of some critical symptoms at various levels of health-care institutions, in order to better detect and react to health-care crises. This would require further development along the lines of MediNet [9] which is a national network of large medical institutions to help in medical information and research data sharing. A well-integrated system with several health-care institutions including general practitioners will help in greater knowledge exchange and integration. Such data may help in any health-care crisis or even bioterrorism preparedness and will provide true flexibility in the context of knowledge integration.

**TABLE I:** Summary of Knowledge Integration Perspective in Managing Health-Care Crisis

Knowledge Integration	Description	Case Study Findings	Implications for Healthcare Information Technologies
Efficiency	Accessing and utilizing specialized knowledge	• Information systems for efficient information flow and communication • Information systems to provide structure for contact tracing operations	Use of information systems capable of quick and efficient sharing of selective information in an emergency to coordinate large scale health crisis management
Scope	Breadth of specialized knowledge drawn upon	• Extend healthcare data scope to help crisis management • Integrating several organizations, sharing domain knowledge towards containing the crisis • Depth of information in the system restricted to contact information, SARS related quarantine information	Involvement of all relevant healthcare and public agencies to share accurate and relevant information pertaining to the crisis. Healthcare information technologies should be adaptable to such utilization. Also, the reach of such information should extend to primary healthcare givers, such as general practitioners.
Flexibility	Accessing additional knowledge and reconfiguring existing knowledge	• Adaptation from other systems indicates ability to add new modules • Potential integrated national health monitoring system • Potential national healthcare knowledge dissemination system	Extending healthcare systems to support efforts to monitor the appearance of epidemics or adapt to new crisis needs. This may also be extended to manage fallouts of bioterrorism.

## VI. Implications and Future Research

Even as medical knowledge expands rapidly, diffusion of such knowledge to clinicians is slow due to numerous barriers [39], hindering their ability to deliver accurate prescriptions and relevant treatment [40]. Knowledge management systems combining content, information science methods, and Internet technologies help structure relevant information from extensive databases such as MEDLINE and CliniWeb [41]. This, in turn, enables provision of better solutions to health-care problems as well as forecast needs and tailors treatment to individual patients based on previously stored information. In the context of a health crisis, knowledge management improves information-sharing and coordination. Similarly, during the early stages of SARS, several hospitals were unable to effectively share information contributing to the spread of the virus. Diagnostic data, alerts, and monitoring of symptoms across clinics are some areas where knowledge management can contribute to better health-care systems (e.g., [40]). However, health-care systems and knowledge practices are at times insufficient to handle a health-care emergency.

Communication infrastructure with multiple agencies to coordinate the management process of a health-care crisis is an important implication for IT in health care arising from this study. There is a strong case for health-care institutions to share information quickly and efficiently when required [7]. HCIS should cater to such needs to prepare for such eventualities in the future, particularly while viral threats [4] and bioterrorist threats [7] abound. The role of IT in enabling such communication and its impact on knowledge-sharing will be an interesting area for future research with useful insights into internal knowledge management, particularly when a task focus is provided to knowledge-sharing activities [3]. Secure data sharing and reliable infrastructure are two important issues arising from the events in Singapore. Often the medical infrastructure in place for medical data sharing [9] provides an effective infrastructure to provide further services. Multilevel data access was implemented by Singapore to secure access to sensitive data.

Health-care crisis response involves multiple agencies [8]. IT can help in structuring and coordinating multiple agency processes during the crisis. E-mail systems can help bring together emergency teams to share a communication medium. E-mail systems can also be made available through multiple platforms including mobile telephony systems, a key connectivity mechanism during the SARS crisis in Singapore. These systems could include all wireless communication enabled devices such as PDAs, etc. Its use and role in health-care IT and knowledge integration should be further investigated.

Cost and technology implications are often a major area of research in health-care-related research [1]. The impact of deploying newer technologies such as XML to make data sharing simpler should be an area of interest, as well the effect of transition to new technologies and the potential need to work in tandem with numerous agencies to effectively manage a large-scale health-care emergency. Expanding the scope of IT and knowledge management in health-care systems is another interesting implication of our study. National health-care symptoms monitoring is an example of potential applications, a useful application in the context of fears of bioterrorism and new strains of viruses. The impact of such systems on medical knowledge-sharing and effectiveness of crisis management will provide useful insights to respective areas of research.

## VII. Conclusion

It is in a position to help organizations manage their health care effectively [1]. A look at the deployment of information systems in Singapore demonstrates the strategic utility of such systems. The system helped MOH focus on information dissemination to address public fear rather than worry about data collection and accuracy. It helped the agencies involved in the process of case management and tracing to focus on quarantining potential patients rather than investigate accuracy of information. The system helped health-care workers focus on giving health care, and streamline information exchange and flow of data necessary for several agencies that managed the outbreak.

As innovations in IT increasingly facilitate knowledge management processes, new opportunities arise for solutions that involve a synergistic combination of a science of information, technology, and knowledge to further enhance the quality of health-care delivery [2]. In a health-care crisis management, such solutions become a necessity. Issues presented in our discussion demonstrate some directions for information systems in health-care management. With the involvement of multiple agencies and their specialized knowledge contributing to the management of the crisis, knowledge integration is a useful perspective. This perspective reveals interesting directions for HCIS in supporting coordination among multiple agencies and sharing knowledge for effective health-care crisis management at a national level.
